# Intersectional analysis of the social vulnerability of individuals with differences in the time between oral cancer diagnosis and treatment: a cross-sectional study, Brazil, 2011-2020

**DOI:** 10.1590/S2237-96222025v34e20240849.en

**Published:** 2025-09-01

**Authors:** João Victor Alves Alencar, Davi Peixoto Craveiro Carvalho, Hebert Garcia Umeno, Mary Anne de Souza Alves França, Monarko Nunes de Azevedo

**Affiliations:** 1 Universidade Federal de Goiás, Faculdade de Medicina, Goiânia, GO, Brazil; 2 Universidade Federal de Goiás, Instituto de Patologia Tropical e Saúde Pública, Departamento de Saúde Coletiva, Goiânia, GO, Brazil

**Keywords:** Mouth Neoplasms, Diagnosis, Therapeutics, Social Vulnerability, Cross-Sectional Studies, Neoplasias de la Boca, Diagnóstico, Terapéutica, Vulnerabilidad Social, Estudios Transversales

## Abstract

**Objective:**

To investigate association between the sociodemographic profiles of individuals with oral cancer in Brazil and the time interval between diagnosis and initiation of treatment.

**Methods:**

This was a cross-sectional study of reported oral cancer cases in Brazil from 2011 to 2020, using data from the Cancer Hospital Registry Information System of the Brazilian National Cancer Institute (Sis-RHC/INCA). Sociodemographic profiles were identified using two-step cluster analysis, and associations were estimated using multinomial logistic regression, with odds ratios (OR) and 95% confidence intervals (95%CI).

**Results:**

The sample included 25,169 cases diagnosed between 2011 and 2020, which defined four distinct profiles: A) married men aged 40-79, White, with less than 8 years of schooling, from the Southern region; B) married men aged 40-79, Black, with 8 years of schooling, from the Northeast region; C) married men aged 40-79, Black, with less than 8 years of schooling, from the Northeast region; D) widowed women aged 40-79, Black, with less than 8 years of schooling, from the Southeast region. Compared to profile D, profile A showed 61.5% lower odds of starting treatment ≥91 days after diagnosis (OR 0.38; 95%CI 0.26; 0.55), profile B showed 50.0% lower odds (OR 0.50; 95%CI 0.32; 0.77), and profile C showed 35.4% lower odds (OR 0.64; 95%CI 0.45; 0.92).

**Conclusion:**

Significant differences exist in the time to treatment initiation for oral cancer across distinct sociodemographic groups.

## Resumen

**Objetivo:**

Verificar la asociación entre los perfiles sociodemográficos de personas con cáncer oral en Brasil y el intervalo entre el diagnóstico y el inicio del tratamiento.

**Métodos:**

Estudio transversal de los casos notificados de cáncer oral en Brasil entre 2011 y 2020, utilizando datos del Sistema de Información de Registros Hospitalarios de Cáncer del Instituto Nacional del Cáncer (Sis-RHC/INCA). Los perfiles sociodemográficos se delinearon mediante un análisis de conglomerados en dos etapas, y las asociaciones se estimaron mediante regresión logística multinomial, utilizando la razón de probabilidades (odds ratio, OR) e intervalos de confianza del 95% (IC95%).

**Resultados:**

La muestra incluyó 25.169 casos diagnosticados entre 2011 y 2020, delineando cuatro perfiles distintos: A) hombres, de 40 a 79 años, blancos, con menos de 8 años de escolaridad, del Sur y casados; B) hombres, 40-79 años, negros, con 8 años de escolaridad, del Nordeste y casados; C) hombres, 40-79 años, negros, con menos de 8 años de escolaridad, del Nordeste y casados; D) mujeres, 40-79 años, negras, con menos de 8 años de escolaridad, del Sudeste y viudas. En comparación con el perfil D, el perfil A presentó una razón de probabilidad un 61,5% menor de iniciar el tratamiento con un intervalo ≥91 días (OR 0,38; IC95% 0,26; 0,55), el perfil B presentó una razón de probabilidad un 50,0% menor (OR 0,50; IC95% 0,32; 0,77) y el grupo C tuvo una probabilidad un 35,4% menor (OR 0,64; IC95% 0,45; 0,92).

**Conclusión:**

Existen diferencias significativas en la forma en que los diferentes grupos sociodemográficos inician el tratamiento contra el cáncer oral.

**Ethical aspects**

This research used public domain anonymized databases.

## Introduction

Noncommunicable chronic diseases pose a major challenge to public health, as they represent the leading cause of death worldwide (1). Among these, malignant neoplasms stand out due to their high incidence, elevated morbidity and mortality, and significant social impact on the quality of life of affected individuals, as well as the high costs of treatment (2). 

Oral cancer is one of the neoplasms of concern in this context, given its negative impact on both physical and psychological well-being (3). In Brazil, 11,029 new oral cancer cases were estimated for the year 2022, including 8,269 in men and 2,760 in women (4). 

From this perspective, several prognostic factors are important in relation to oral cancer, including patient age and tumor location, thickness, and staging. Thus, early and accurate diagnosis and treatment are essential for a better prognosis, as they enable the disease to be addressed in less advanced stages (5). 

Accordingly, late identification and delayed treatment initiation are factors that worsen prognosis for these patients (5). Hence, the importance of implementing Law No. 12732/2012, which establishes a maximum interval of 60 days between cancer diagnosis and treatment initiation, including the oral type (6). 

A study revealed that in Brazil, since 2018, there has been a significant reduction in the time delay for the initiation of oral cancer treatment in most cases (7). However, when examining compliance with the legal 60-day time frame, most regions of the country still show a stationary trend, denoting disparity between the Brazilian macro-regions (7). 

Despite legal progress, social vulnerabilities continue to significantly impact the interval between diagnosis and treatment initiation across different types of cancer (8). Understanding this issue requires investigating how the intersection of social vulnerabilities influences the experience of patients with oral cancer. Intersectionality refers to the overlapping of social identities—such as race/skin color, gender, and class—which generate inequalities in health care access and cancer outcomes (9). In oral cancer, this approach helps explain why Black individuals face greater difficulties accessing treatment and lower post-diagnosis survival rates (10). Additionally, patients from regions with high mortality may be exposed to under-resourced health systems and structural barriers that compromise prognosis (11).

Despite the relevance of a comprehensive, intersectional analysis of treatment-related aspects in different types of cancer, there remains a significant knowledge gap concerning oral cancer—particularly in identifying vulnerable groups facing the greatest difficulties in accessing timely treatment. 

This study aimed to investigate association between sociodemographic profiles of individuals with oral cancer in Brazil and the interval between diagnosis and treatment initiation.

## Methods

### Design

This was a cross-sectional study using secondary data on oral cancer cases registered in Brazil between 2011 and 2020.

### Participants

The study population consisted of individuals of all ages and sexes with confirmed diagnosis of oral cancer, classified as C00 to C06 according to the 10th revision of the International Classification of Diseases – ICD-10 (12).

Cases with missing variables needed for analysis or those with a negative time interval between diagnosis and treatment (i.e., surgery performed before histopathological diagnosis) were excluded, as they were not appropriate for the proposed analysis.

### Variables

The dependent variables were the interval between diagnosis and treatment—defined as the number of days between histopathological confirmation and treatment initiation—and cancer staging at diagnosis, based on the tumor-node-metastasis (TNM) classification system (13). The interval was categorized as 0-60, 61-90, and ≥91 days. Staging was categorized as carcinoma in situ (0) and stages I, II, III, and IV, according to combinations of T, N, and M categories (13). 

The independent variables were sex (male and female); age (continuous and age group, in years [0-39, 40-79, and 80 or older]); race/skin color (White, Black, and other); education (less than 8 years, 8 years, 11 years, and 15 years or more); marital status (single, married/common-law marriage, widowed, and divorced); treatment funding (Brazilian National Health System [*Sistema Único de Saúde* - SUS] and health insurance plan/out-of-pocket costs); and region of the country (North, Northeast, Southeast, South, and Midwest)

Categorical variables were described using absolute and relative frequencies. For age (continuous), mean, median, and standard deviation were calculated.

### Data sources and measurement

Data were obtained from the Cancer Hospital Registry Information System of the Brazilian National Cancer Institute (Sis-RHC/INCA), which includes sociodemographic, clinical, and treatment characteristics (https://irhc.inca.gov.br/RHCNet/selecionaTabulador.action?initial=1&local=todosho_sp&unidFed=) (14). 

This database was chosen for its inclusion of variables enabling analysis of intersectionality between social vulnerability and oral cancer (15). 

### Bias control

Data completeness and consistency were assessed by identifying missing values and temporal inconsistencies. 

### Study Size

All available records for the study period were included, ensuring sample representativeness. Random sampling was not applied since the secondary data covered a significant portion of the target population.

### Statistical methods

Associations between each independent variable and the diagnosis-to-treatment interval were tested using Pearson’s chi-square test. Then, multinomial logistic regression was applied to estimate the strength of association for each independent predictor, using the 0-60 day category as the reference.

The profiles were outlined using two-step cluster analysis, so as to group cases or objects according to the degree of similarity observed between them. Sociodemographic variables were used, since they help describing groups of individuals that are internally homogeneous yet different from each other according to these characteristics. In the model, the following variables were used: sex, age, race/skin color, education level, treatment funding, marital status, and region. 

The method used was the two-step cluster analysis, available in the Statistical Package for Social Science for Windows (SPSS 23.0). This is recommended for analyzing data with continuous and categorical variables. 

It works based on a sequence of agglomerative partitions. First, pre-clusters were formed, which could be individual cases or small groups. In the second step, the pre-clusters were regrouped, forming final subprofiles, according to an ideal number of clusters. To define the best number of clusters, the Bayesian information criterion (BIC) was applied, and the distance measure used was log-likelihood, both being standard options of the program.

The sociodemographic profiles were described and compared with regard to the variables “diagnosis-to-treatment interval” and “cancer staging at diagnosis” by analyzing the difference in proportions using Pearson’s chi-square or Fisher’s exact test with Bonferroni correction, with a 0.05 significance level.

Multinomial logistic regression was used to assess association between the identified profiles and the diagnosis-to-treatment interval, independent of cancer staging, with a 0.05 significance level. Odds ratios (OR) were calculated to quantify the strength of associations, along with 95% confidence intervals (95% CI) to assess the accuracy of estimates. 

The 0-60 day category was used as the reference in the regression models, as it corresponds to the legally mandated maximum interval for treatment initiation, according to Law No. 12732/2012 (6).

### Data access and cleaning methods 

The dataset used for analysis, including codes and relevant data, is available from the SciELO Data repository (https://doi.org/10.48331/scielodata.PLDF5O) (16). Microsoft Excel was used for data tabulation.

### Data Matching

The study relied on a single dataset, and therefore data matching with other sources was not necessary. Data integrity was validated internally through manual review of random samples. Variables were formatted, standardized, and categorized according to the study criteria, with categorical variables grouped to ensure uniformity and consistency in statistical analysis.

## Results

A total of 67,257 records of oral cancer were identified on the Sis-RHC/INCA database. Initially, 13,358 cases (19.9%) were excluded due to lack of information on the interval between diagnosis and treatment, so that 53,899 cases remained (80.1%). Next, 2,890 cases (4.3%) with a negative interval were removed, leaving a total of 51,009 cases (75.8%). Finally, 25,840 cases (38.4%) with at least one missing independent variable were excluded, leading to a total of 25,169 cases (37.4%) included in the analysis (Supplementary Figure 1).

The mean age of the 25,169 cases was 60.1 years, standard deviation was 12.7 years, and the median was 60 years. Most individuals presented the following characteristics: 40-79 years old (88.5%), male (75.3%), Black or Brown skin color (56.2%), less than 8 years of schooling (66.1%), residing in the Southeast region (37.6%), with treatment covered by the SUS (85.8%), and married/common-law marriage (50.2%). Less than half of the individuals with oral cancer (43%) began treatment within 60 days after diagnosis, and most cases were diagnosed at stage IV (37.1%) (Table 1). The mean and median intervals between diagnosis and treatment were 95.9 and 70 days, respectively.

**Table 1:** Sociodemographic and clinical characteristics of patients with oral cancer. Brazil, 2011-2020 (n=25,169)

Variables	n (%)
**Age group** (years)	
0-39	1,138 (4.6)
40-79	22,290 (88.5)
80 or older	1,741 (6.9)
Sex	
Male	18,957 (75.3)
Female	6,212 (24.7)
**Race/skin color**	
White	10,849 (43.1)
Black	14,143 (56.2)
Other	177 (0.7)
**Education level**	
Less than 8 years	16,636 (66.1)
8 years	4,382 (17.4)
11 years	3,385 (13.4)
15 years or more	766 (3.0)
Region	
North	1,128 (4.5)
Northeast	7,618 (30.3)
Southeast	9,465 (37.6)
South	6,355 (25.2)
Midwest	603 (2.4)
**Treatment funding**	
SUS	21,589 (85.8)
Health insurance plan/out-of-pocket expense	3,580 (14.2)
**Marital status**	
Single	7,376 (29.3)
Married/common-law marriage	12,628 (50.2)
Widowed	2,777 (11.0)
Divorced	2,388 (9.5)
**Diagnosis-to-treatment interval** (days)	
0-60	10,826 (43.0)
61-90	5,001 (19.9)
≥91	9,342 (37.1)
**Cancer staging at diagnosis**	
In situ (0)	23 (0.1)
Stage I	1,372 (5.5)
Stage II	2,021 (8.0)
Stage III	3,127 (12.4)
Stage IV	9,348 (37.1)
Not informed	9,278 (36.9)

All variables analyzed individually remained associated with the time interval between diagnosis and treatment (Table 2). When observing the odds of starting treatment within 91 days or more after diagnosis for each independent variable, the following patterns emerged: men were 7.8% less likely to experience delay compared to women (OR 0.92; 95%CI 0.86; 0.99); individuals aged 0-39 were 18.3% less likely to experience delay compared to those aged 40-79 (OR 0.82; 95%CI 0.71; 0.94); White individuals were 10.9% less likely to experience delay than Black individuals (OR 0.89; 95%CI 0.83; 0.96); those with 15 or more years of education were 36.6% less likely to experience delay than those with less than 8 years (OR 0.63; 95%CI 0.53; 0.76); individuals from the Southern region were 52.3% less likely to experience delay than those from the Southeast (OR 0.48; 95%CI 0.44; 0.51); and patients whose treatment was an out-of-pocket expense or funded through health insurance plan were 27.6% less likely to experience delay compared to those whose treatment was covered by SUS (OR 0.72; 95%CI 0.67; 0.79) (Supplementary Table 1).

**Table 2:** Association between independent variables and the diagnosis-to-treatment interval. Brazil, 2011-2020 (n=25,169)

Variables	0-60 days n (%)	61-90 days n (%)	≥91 days n (%)	p-value
**Age group** (years)				0.033
0-39	533 (46.8)	228 (20.0)	377 (33.1)	
40-79	9,532 (42.8)	4,420 (19.8)	8,338 (37.4)	
80 or older	761 (43.7)	353 (20.3)	627 (36.0)	
Sex				0.001
Male	8,184 (43.2)	3,843 (20.3)	6,930 (36.6)	
Female	2,642 (42.5)	1,158 (18.6)	2,412 (38.8)	
**Race/skin color**				<0.001
White	5,157 (47.5)	1,983 (18.3)	3,709 (34.2)	
Black	5,571 (39.4)	2,990 (21.1)	5,582 (39.5)	
Other	98 (55.4)	28 (15.8)	51 (28.8)	
**Education level**				<0.001
More than 8 years	6,803 (40.9)	3,344 (20.1)	6,489 (39.0)	
8 years	2,019 (46.1)	860 (19.6)	1,503 (34.3)	
11 years	1,586 (46.9)	662 (19.6)	1,137 (33.6)	
15 years or more	418 (54.6)	135 (17.6)	213 (27.8)	
Region				<0.001
North	434 (38.5)	198 (17.6)	496 (44.0)	
Northeast	3,308 (43.4)	1,479 (19.4)	2,831 (37.2)	
Southeast	3,379 (35.7)	2,135 (22.6)	3,951 (41.7)	
South	3,367 (53.0)	1,109 (17.5)	1,879 (29.6)	
Midwest	338 (56.1)	80 (13.3)	185 (30.7)	
**Treatment funding**				<0.001
SUS	9,039 (41.9)	4,324 (20.0)	8,226 (38.1)	
Health insurance plan/out-of-pocket expense	1,787 (49.9)	677 (18.9)	1,116 (31.2)	
**Marital status**				<0.001
Single	3,011 (40.8)	1,469 (19.9)	2,896 (39.3)	
Married/common-law marriage	5,624 (44.5)	2,475 (19.6)	4,529 (35.9)	
Widowed	1,173 (42.2)	550 (19.8)	1,054 (38.0)	
Divorced	1,018 (42.6)	507 (21.2)	863 (36.1)	

In the two-step cluster analysis, the variables with the greatest predictive importance regarding profile were: sex, race/skin color, education level, marital status, age group, and region. Treatment funding showed the least weight in defining the profiles. Four distinct sociodemographic profiles were identified (Table 3):

**Table 3:** Frequency of sociodemographic characteristics according to the profiles of patients with oral cancer. Brazil, 2011-2020 (n=25,169)

Sociodemographic characteristics	Profile A 39.1% (9,843)	Profile B 16.3% (4,108)	Profile C 32.4% (8,150)	Profile D 12.2% (3,068)
Sex				
Male	81.6	76	78	47.4
Female	18.4	24	22	52.6
**Age group** (years)				
0-39	10	12	4	0.2
40-79	80	85	81	60
80 or older	10	3	15	39.8
**Race/skin color**				
White	96.9	1.65	0	40.7
Black/Brown	2.1	96.7	99.7	59.3
Other	1	1.65	0.3	0
**Education level** (years)				
Less than 8	59.7	0	100	85.1
8	18.2	53.6	0	13.9
11	15	40	0	1
15 or more	7.1	6.4	0	0
Region				
North	0.5	5.9	7	4.5
Northeast	8.1	43.3	48.6	33
Southeast	39.4	39.5	38	35.5
South	51.5	5.9	4	25
Midwest	0.5	5.3	2.4	2
**Marital status**				
Single	22.5	30	44.6	0
Married/common-law marriage	61.4	50.2	55.4	0
Widowed	0	8.8	0	84.3
Divorced	16.1	11	0	15.7

Profile A – predominantly White men (96.9%) with less than 8 years of schooling (59.7%), residing in the Southern region (51.5%), married/common-law marriage (61.4%), aged 40-79; Profile B – Black men (96.7%) with 8 years of schooling (53.6%), from the Northeast (43.3%), married/common-law marriage (50.2%), aged 40-79; Profile C – Black men (99.7%) with less than 8 years of schooling (100%), from the Northeast (48.6%), married/common-law marriage (55.4%), aged 40-79; Profile D – Black women (59.3%) with less than 8 years of schooling (85.1%), from the Southeast (35.5%), widowed (84.3%), aged 40-79 (Table 3).

The majority of individuals in Profile A (52.8%) started treatment within 0-60 days after diagnosis. Less than half of those in Profiles B (43.7%), C (40.9%), and D (31.4%) began treatment within this time frame. Profile D stood out for having the highest proportion of patients (46.9%) who started treatment ≥91 days after diagnosis (p-value<0.001) (Table 4).

**Table 4:** Comparison of sociodemographic profiles of patients with oral cancer, according to the diagnosis-to-treatment interval and staging. Brazil, 2011-2020 (n=25,169)

Sociodemographic characteristics	Profile A^a^	Profile B^b^	Profile C^c^	Profile D^d^	p-value
**Diagnosis-to-treatment interval** (days)					<0.001
0-60	52.8	43.7	40.9	31.4	
61-90	16.8	23.7	19.7	21.7	
≥91	30.3	32.6	39.4	46.9	
Staging					0.005
In situ (0) and stage I	11.4	7.3	7.0	15.4	
Stage II	12.3	16.9	13.1	13.1	
Stages III and IV	76.3	75.8	79.9	71.5	

^a^Profile A men aged 40-79, White, with less than 8 years of schooling, from the South region, and married; ^b^Profile B:men aged 40-79, Black, with 8 years of schooling, from the Northeast region, and married; ^c^Profile C: men aged 40-79, Black, with less than 8 years of schooling, from the Northeast region, and married; ^d^Profile D: women aged 40-79, Black, with less than 8 years of schooling, from the Southeast region, and widowed.

The proportion of individuals diagnosed at stages III and IV was high across all profiles:

Profile A (76.3%), B (75.8%), C (79.9%), and D (71.5%) showed similar rates. For early stages (0 and I), Profiles A (11.4%) and D (15.4%) had higher proportions compared to B (7.3%) and C (7.0%). At stage II, proportions were similar across all profiles: A (12.3%), B (16.9%), C (13.1%), and D (13.1%) (p-value 0.005) (Table 4).

Compared to Profile D, individuals in Profiles A, B, and C showed different odds of starting treatment within 61-90 days and ≥91 days. The associations between Profiles B and C and the 61-90 day interval were not statistically significant. However, Profile A remained associated with lower odds of treatment within 61-90 days. All profiles were significantly associated with treatment ≥91 days after diagnosis:

Profile A had 61.5% lower odds compared to Profile D (OR 0.38; 95%CI 0.26; 0.55); Profile B had 50% lower odds (OR 0.50; 95%CI 0.32; 0.77); Profile C had 35.4% lower odds (OR 0.64; 95%CI 0.45; 0.92) (Table 5).

**Table 5:** Odds ratio (OR) and 95% confidence intervals (95% CI) for the diagnosis-to-treatment interval and staging, according to the profiles of patients with oral cancer. Brazil, 2011-2020 (n=25,169)

Profiles	61-90 days OR (95%IC)	p-value	≥91 days OR (95%IC)	p-value
White men with lower levels of education, from the Southern region, with a significant other	0.46 (0.29; 0.71)	0.001	0.38 (0.26; 0.55)	<0.001
Black men with higher levels of education, from the Northeast region, with a significant other	0.78 (0.47; 1.30)	0.348	0.50 (0.32; 0.77)	0.002
Black men with lower levels of education, from the Northeast region, with a significant other	0.69 (0.45; 1.08)	0.107	0.64 (0.45; 0.92)	0.016
Black women with lower levels of education, from the Southeast region, that are widowed	1.00	__	1.00	__

## Discussion

This study aimed to analyze inequalities in the time between diagnosis and initiation of treatment for oral cancer in Brazil, with a focus on social markers of difference from an intersectional perspective. The intersectional analysis revealed disparities in access: patients in Profile A (White men from the South with better socioeconomic conditions) started treatment more quickly, while those in Profile D (Black women from the Southeast with low education levels) were more likely to experience delays.

The study identified statistical differences between some characteristics of the patients included in the final analysis and those excluded. Using a database with a high proportion of missing variables limits the generalizability of the results, which should be attributed only to the analyzed population and not necessarily represents all patients undergoing treatment for oral cancer in Brazil during the study period (17). 

There was a predominance of diagnoses at advanced stages (III and IV), which reduces the odds of successful treatment and worsens prognosis (18). This pattern of late diagnosis indicates shortcomings in the health system in terms of screening and early detection, suggesting a lack of nationally institutionalized oral cancer awareness and screening programs (19). This may be due to several challenges, such as a lack of educational campaigns, barriers to accessing primary health care, and insufficient training of health professionals to recognize early signs of the disease (19).

Delays in starting treatment for oral cancer are another significant problem, as they reduce cure rates, worsen prognosis, and increase health system costs (7). Despite the provisions of Law No. 8080/1990, fewer than half of patients began treatment within the recommended 60 days, showing that delays are still prevalent. This delay reflects a lack of coordination between levels of care and logistical difficulties in referral processes. It highlights the need for strategies to strengthen oncology care pathways, including integration between services, active surveillance, efficient regulation, and expansion of capacity at referral centers (20).

Higher prevalence of oral cancer was observed among men, suggesting the influence of sex in disease occurrence, possibly related to greater exposure to risk factors such as smoking, alcohol use, hazardous occupational environments, and lower adherence to preventive practices influenced by masculinity norms (4,21). Regarding age, the proximity between mean and median (60 years) points to a symmetrical distribution, emphasizing that oral cancer primarily affects individuals around 60 years old. This is consistent with the epidemiological profile described in the literature, which associates the disease with cumulative exposure over the course of life (4). This age pattern stresses the importance of public policies for prevention, screening, and early diagnosis in the elderly population, especially in aging contexts like Brazil (22) 

Each independent variable was found to be a predictor of the time between diagnosis and treatment initiation, with greater delays observed among women, individuals aged 40-79, Black individuals, those with low education, residents of the Southeast region, and patients covered by the SUS Similarly, a retrospective cohort of over 79,000 colorectal cancer cases (2006-2015) showed greater delays among patients over 50, Black individuals, and those with low education (23). Likewise, a retrospective cohort of 137,593 women with breast cancer recorded on the Sis-RHC (2000-2011) identified greater vulnerability to delays among older women, non-White women, unmarried women, women with low education, and those covered by SUS (24). 

Nevertheless, although individual variables have influenced the time between diagnosis and the start of treatment for oral cancer, it is essential to analyze the impact of sociodemographic profiles on the time until treatment begins, from the perspective of the intersectionality of these independent variables. Review studies indicate that policies focused on only one dimension of identity (such as sex or race/skin color alone) fail to capture the complexity of individual experiences, while incorporating intersectionality allows for the development of more effective strategies against systemic inequalities (25). 

The intersectional analysis revealed that Black women from the Southeast, aged 40-79, widowed, and with low education levels experienced greater delays in treatment initiation, indicating vulnerabilities associated with the overlap of social markers. This pattern is consistent with findings from a similar cross-sectional study conducted in Belo Horizonte (Minas Gerais, Brazil) with 715 women with breast cancer registered on Sis-RHC (2010-2013), in which Black women with less than 8 years of schooling and covered by SUS were more likely to begin breast cancer treatment after 91 days compared to White, more educated women treated in the private sector (17). This result shows that the greater the level of social vulnerability among women, the greater the likelihood of delay (p-value<0.001) (17). In that previous study, marital status had no importance regarding profile prediction (17), whereas in the present study, the variable with the least weight was treatment funding. 

These social vulnerabilities impact treatment initiation in different ways and, when intersected, result in the delays observed in Profile D. White married men from the South, represented by Profile A, tend to have quicker access to treatment due to better educational conditions, less discrimination, and a more developed regional health system (7). In contrast, Black widowed women from the Southeast (Profile D) face multiple barriers stemming from the intersectionality of these variables, which hinders timely access to treatment (17,26). Absence of a significant other is associated with greater delays in diagnosis, possibly due to reduced access to preventive care and greater financial and family burden (27). Married women tend to seek care earlier, benefitting from their partner’s support in identifying and discussing symptoms (28). Higher education levels also facilitate communication with health professionals and symptom reporting, improving navigation through the health system for diagnosis and treatment initiation (29).

This study has several limitations that should be considered. The aggregated nature of the data limits individualized analyses and precludes causal inferences (18). It is important to emphasize that this is one of the first studies to address the time between diagnosis and treatment initiation for oral cancer from an intersectional perspective, highlighting the need for future research using other databases and regional breakdowns to deepen the understanding of social vulnerabilities involved (7). Potential clinical confounding factors, such as infection with human papillomavirus type 16 (HPV-16), sun exposure, and obesity, were not accounted for due to the absence of such information in the database. A significant limitation lies in the quality of data entry in the Sis-RHC/INCA system, including underreporting and regional discrepancies that may affect the accuracy of the estimates. These issues are influenced by factors such as infrastructure, workforce training, and data entry standardization (30), which poses the need for a cautious interpretation of the study’s findings. Even so, data quality has improved over the years, reinforcing the importance of its continuous enhancement for cancer epidemiological surveillance and oncological care planning (17,30). 

The analysis of sociodemographic profiles shows that intersectionality deepens the understanding of inequalities in access to healthcare. It highlights the need for public policies that take these interactions into account to improve timely access to cancer treatment for vulnerable groups and to ensure the application of the SUS principles of comprehensiveness, universality, and equity to the reality of all individuals (7).

To conclude, Black women from the Southeast region, aged 40 to 79, with low education levels and widowed were more likely to begin treatment more than 91 days after diagnosis. Although most cases were diagnosed at advanced stages (III and IV), there were significant disparities in how different sociodemographic groups accessed and initiated oral cancer treatment. 

## Data Availability

The entire dataset supporting the results of this study has been made available on SciELO Data and can be accessed at https://doi.org/10.48331/scielodata.PLDF5O.

## Supplementary material

Supplementary Figure 1, Supplementary Table 1.
