# Supplementary material for: Intersectional analysis of the social vulnerability of individuals with differences in the time between oral cancer diagnosis and treatment: a cross-sectional study, Brazil, 2011-2020
Source: Epidemiol Serv Saude. 2025 Sep 1;34:e20240849. doi: 10.1590/S2237-96222025v34e20240849.en (PMC12404597; doi:10.1590/S2237-96222025v34e20240849.en)
Supplement: Supplementary file 1 [file 2237-9622-ress-34-e20240849-suppl01-en.pdf]

Supplementary Figure 1– Multi-stage case selection and exclusion process

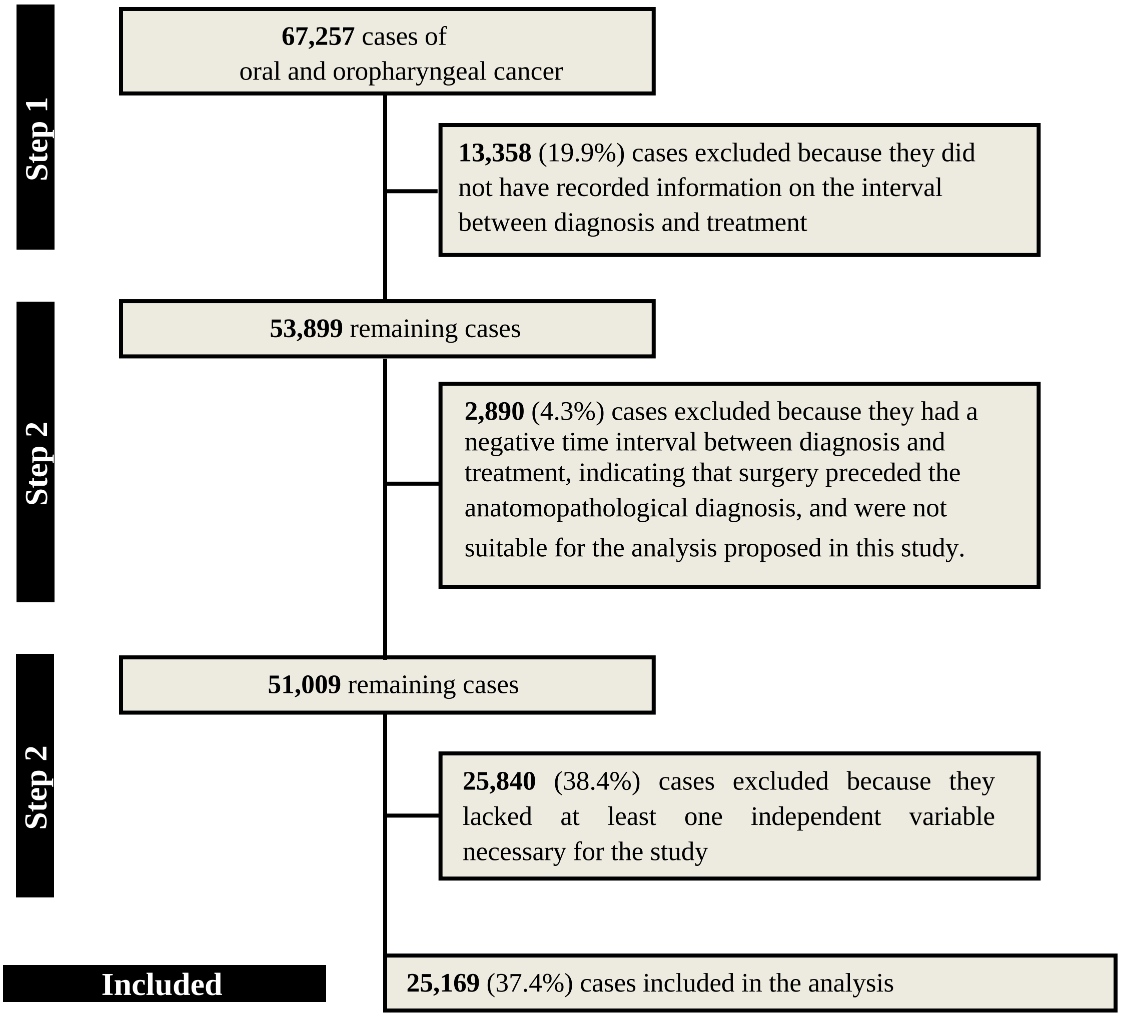

Supplementary Table 1 – Odds ratio (OR) and confidence intervals (95%CI) for the time interval between diagnosis and treatment according to each independent variable. Brazil, 2011-2020 (n=25,169)

	61 to 90 days interval	≥ 91-days interval
	OR (95%IC)	OR (95%IC)
**Sex**		
**Male**	1.08 (0.99; 1.17)	0.92 (0.86; 0.99)
**Female**	1.00	1.00
**Age group (years)**		
**0-39**	0.96 (0.82; 1.14)	082 (0.71; 0.94)
**80 or older**	1.01 (0.88; 1.16)	0.90 (0.80; 1.01)
**40-79**	1.00	1.00
**Race/skin color**		
**White**	0.79 (0.73; 0.86)	0.89 (0.83; 0.96)
**Other**	0.61 (0.40; 0.94)	0.63 (0.44; 0.88)
**Black**	1.00	1.00
**Education level**		
**8 years**	0.90 (0.82; 0.99)	0.82 (0.76; 0.88)
**11 years**	0.89 (0.80; 0.99)	0.80 (0.73; 0.87)
**15 years or more**	0.76 (0.62; 0.94)	0.63 (0.53; 0.76)
**Less than 8 years**	1.00	1.00
**Region**		
**Central-West**	0.38 (0.29; 0.48)	0.47 (0.39; 0.56)
**Northeast**	0.71 (0.65; 0.77)	0.73 (0.68; 0.78)
**North**	0.72 (0.61; 0.86)	0.98 (0.85; 1.12)
**South**	0.52 (0.48; 0.57)	0.48 (0.44; 0.51)
**Southeast**	1.00	1.00
**Treatment funding**		
**Health insurance plan/Out-of-the-pocket expense**	0.83 (0.75; 0.91)	0.72 (0.67; 0.79)
**SUS**	1.00	1.00

Marital status		
**Single**	0.98 (0.86; 1.11)	1.05 (0.94; 1.17)
**Married/Common-law marriage**	0.94 (0.84; 1.06)	0.95 (0.86; 1.05)
**Divorced**	1.06 (0.91; 1.24)	1.00 (0.88; 1.14)
**Widowed**	1.00	1.00
