# Supplementary material for: Intersectional analysis of the social vulnerability of individuals with differences in the time between oral cancer diagnosis and treatment: a cross-sectional study, Brazil, 2011-2020
Source: Epidemiol Serv Saude. 2025 Sep 1;34:e20240849. doi: 10.1590/S2237-96222025v34e20240849.en (PMC12404597; doi:10.1590/S2237-96222025v34e20240849.en)
Supplement: Supplementary file 2 [file 2237-9622-ress-34-e20240849-suppl01-pt.pdf]

Figura Suplementar 1 – Processo de seleção e exclusão dos casos em múltiplas etapas

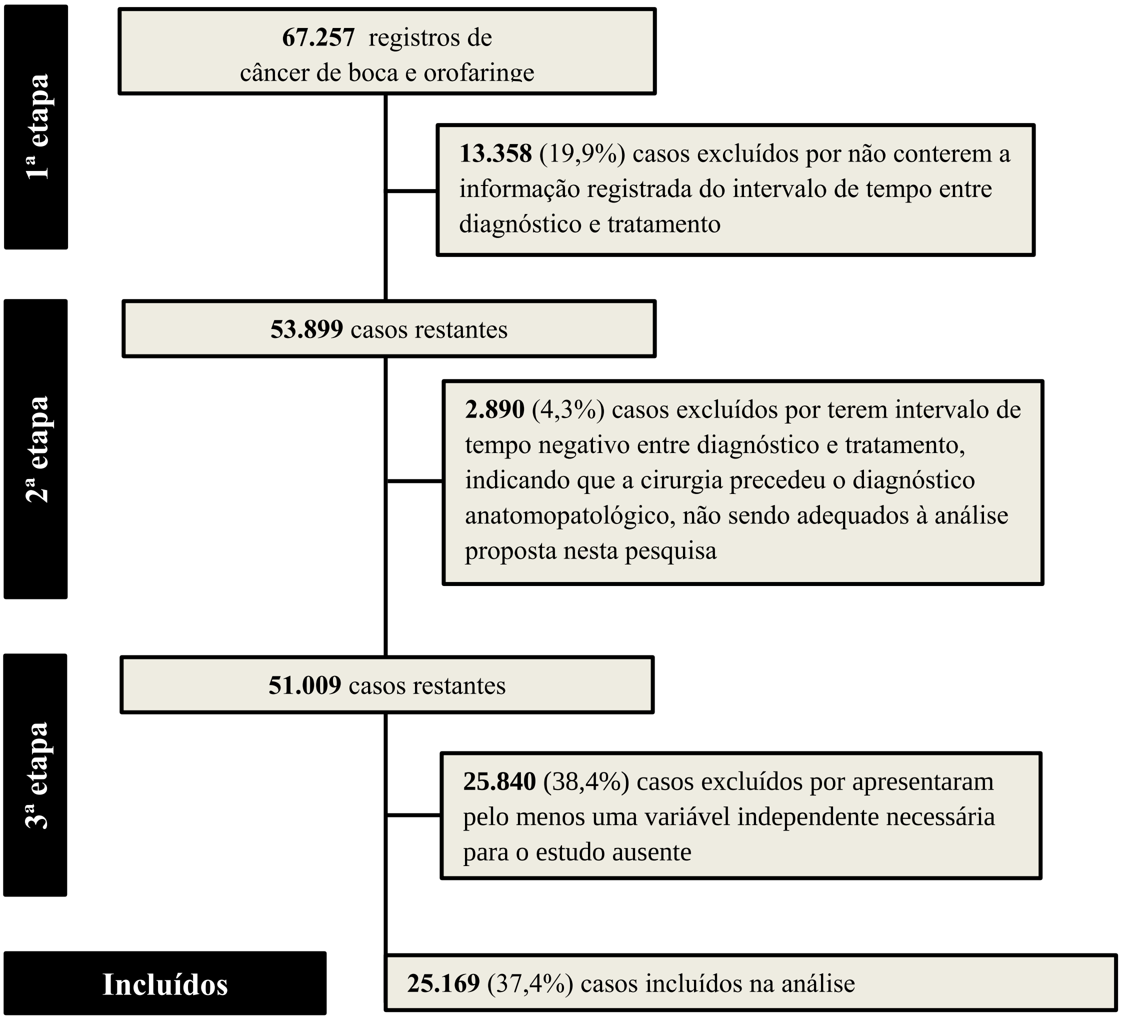

Tabela Suplementar 1 – Razão de chances (*odds ratio*, OR) e Intervalos de Confiança (IC95%) do intervalo de tempo entre diagnóstico e tratamento de acordo com cada variável independente. Brasil, 2011-2020 (N = 25169)

	**Intervalo 61 a 90 dias OR (IC95%)**	**Intervalo ≥ 91 dias OR (IC95%)**
**Sexo**		
**Masculino**	1,08 (0,99; 1,17)	0,92 (0,86; 0,99)
**Feminino**	1,00	1,00
**Faixa etária (anos)**		
**0-39**	0,96 (0,82; 1,14)	082 (0,71; 0,94)
**80 ou mais**	1,01 (0,88; 1,16)	0,90 (0,80; 1,01)
**40-79**	1,00	1,00
**Raça/Cor da pele**		
**Brancos**	0,79 (0,73; 0,86)	0,89 (0,83; 0,96)
**Outros**	0,61 (0,40; 0,94)	0,63 (0,44; 0,88)
**Negros**	1,00	1,00
**Escolaridade**		
**8 anos**	0,90 (0,82; 0,99)	0,82 (0,76; 0,88)
**11 anos**	0,89 (0,80; 0,99)	0,80 (0,73; 0,87)
**15 anos ou mais**	0,76 (0,62; 0,94)	0,63 (0,53; 0,76)
**Menor que 8 anos**	1,00	1,00
**Região**		
**Centro-Oeste**	0,38 (0,29; 0,48)	0,47 (0,39; 0,56)
**Nordeste**	0,71 (0,65; 0,77)	0,73 (0,68; 0,78)
**Norte**	0,72 (0,61; 0,86)	0,98 (0,85; 1,12)
**Sul**	0,52 (0,48; 0,57)	0,48 (0,44; 0,51)
**Sudeste**	1,00	1,00
**Custeio do tratamento**		
**Plano de saúde/particular**	0,83 (0,75; 0,91)	0,72 (0,67; 0,79)
**SUS**	1,00	1,00

**Estado conjugal**		
**Solteiro**	0,98 (0,86; 1,11)	1,05 (0,94; 1,17)
**Casado/união consensual**	0,94 (0,84; 1,06)	0,95 (0,86; 1,05)
**Separado**	1,06 (0,91; 1,24)	1,00 (0,88; 1,14)
**Viúvo**	1,00	1,00
