# Supplementary material for: Intersectional analysis of the social vulnerability of individuals with differences in the time between oral cancer diagnosis and treatment: a cross-sectional study, Brazil, 2011-2020
Source: Epidemiol Serv Saude. 2025 Sep 1;34:e20240849. doi: 10.1590/S2237-96222025v34e20240849.en (PMC12404597; doi:10.1590/S2237-96222025v34e20240849.en)
Supplement: Translation

# Translation: Análise interseccional da vulnerabilidade social de pessoas com diferenças no intervalo entre o diagnóstico e o tratamento do câncer de boca: estudo transversal, Brasil, 2011-2020

**DOI:** 10.1590/S2237-96222025v34e20240849.pt

**Authors:** João Victor Alves Alencar, Davi Peixoto Craveiro Carvalho, Hebert Garcia Umeno, Mary Anne de Souza Alves França, Monarko Nunes de Azevedo

**Affiliations:** 1 Universidade Federal de Goiás, Faculdade de Medicina, Goiânia, GO, Brasil; 2 Universidade Federal de Goiás, Instituto de Patologia Tropical e Saúde Pública, Departamento de Saúde Coletiva, Goiânia, GO, Brasil

**Keywords:** Neoplasias Bucais, Diagnóstico, Terapêutica, Vulnerabilidade Social, Estudos Transversais

## Abstract

**Objetivo:**

Verificar a associação entre os perfis sociodemográficos de indivíduos com câncer de boca no Brasil e o intervalo entre diagnóstico e início do tratamento.

**Métodos:**

Estudo transversal dos casos notificados de câncer de boca no Brasil de 2011 a 2020, utilizando dados do Sistema de Informação de Registros Hospitalares de Câncer do Instituto Nacional de Câncer (Sis-RHC/INCA). Os perfis sociodemográficos foram delineados por análise de aglomerados em duas etapas, e as associações foram estimadas por regressão logística multinomial, utilizando razão de chances (*odds ratio*, OR) e intervalos de confiança de 95% (IC95%).

**Resultados:**

A amostra incluiu 25.169 casos diagnosticados entre 2011 e 2020, delineando quatro perfis distintos: A) homens, 40-79 anos, brancos, com escolaridade menor que 8 anos, do Sul e casados; B) homens, 40-79 anos, negros, com escolaridade de 8 anos, do Nordeste e casados; C) homens, 40-79 anos, negros, com escolaridade menor que 8 anos, do Nordeste e casados; D) mulheres, 40-79 anos, negras, com escolaridade menor que 8 anos, do Sudeste e viúvas. Comparados ao perfil D, A apresentou razão de chances 61,5% menor de iniciar o tratamento com intervalo ≥91 dias (OR 0,38; IC95% 0,26; 0,55), B teve razão de chances 50,0% menor (OR 0,50; IC95% 0,32; 0,77), e C teve razão de chances 35,4% menor (OR 0,64; IC95% 0,45; 0,92).

**Conclusão:**

Existem diferenças significativas na forma como diferentes grupos sociodemográficos iniciam o tratamento para câncer de boca.

**Aspectos éticos**

Esta pesquisa utilizou bancos de dados de domínio público e anonimizados.

## Introdução

As doenças crônicas não transmissíveis representam grande desafio para a saúde pública, por constituírem a principal causa de óbitos no mundo (1). Dentre elas, as neoplasias malignas se destacam devido à alta incidência, à elevada morbimortalidade e ao impacto social na qualidade de vida das pessoas acometidas pela doença, além dos altos custos de tratamento (2). 

O câncer de boca é uma das neoplasias que têm relevância nesse aspecto, por impactar negativamente a qualidade de vida das pessoas acometidas pela doença, tanto no aspecto físico como no psicológico (3). No Brasil, em 2022, a estimativa foi de 11.029 casos por ano, sendo 8.269 homens e 2.760 mulheres (4). 

Sob essa ótica, há alguns fatores prognósticos importantes em relação ao câncer de boca, relativos à idade do paciente, localização, espessura e classificação do tumor. Por isso, o diagnóstico e o tratamento precoce e acurado são essenciais para melhor prognóstico, visto que possibilitam a abordagem do tumor em estágio de menor gravidade (5). 

Desse modo, observa-se que a identificação tardia e o atraso no início do tratamento são fatores que pioram o prognóstico para esses pacientes (5). Assim, entende-se a importância da implementação da Lei n^o^ 12.732/2012, que estabelece intervalo de no máximo 60 dias entre o diagnóstico e o início do tratamento do câncer, incluindo o câncer de boca (6). 

Estudo revelou que, desde 2018, vem sendo percebida redução significativa do tempo de demora para o início do tratamento do câncer de boca na maioria dos casos no Brasil (7). No entanto, quando analisada a tendência do tempo máximo estabelecido pela legislação (60 dias), a maioria das regiões do país ainda apresentam tendência estacionária, denotando disparidade entre as macrorregiões brasileiras (7). 

Mesmo com os avanços legais, as vulnerabilidades sociais ainda impactam significativamente o tempo entre diagnóstico e início do tratamento em diferentes neoplasias (8). Compreender melhor esse problema exige entender a influência da interseccionalidade dessas vulnerabilidades na experiência dos pacientes com câncer de boca. A interseccionalidade refere-se à sobreposição de identidades sociais, como raça/cor da pele, gênero e classe, que geram desigualdades no acesso à saúde e nos desfechos oncológicos (9). No câncer de boca, esse enfoque contribui para compreender por que indivíduos negros enfrentam maior dificuldade no acesso a tratamentos e menores taxas de sobrevivência pós-diagnóstico (10). Além disso, pacientes de regiões com alta mortalidade também podem estar expostos a sistemas de saúde precários e barreiras estruturais que comprometem o prognóstico (11).

Apesar da importância de uma análise abrangente e interseccional dos aspectos relacionados ao tratamento das neoplasias em diferentes tipos de câncer, há ainda lacuna significativa de conhecimento para o câncer de boca, especialmente no detalhamento dos grupos vulneráveis com maiores dificuldades para o início do tratamento. 

O objetivo deste estudo foi verificar a associação entre os perfis sociodemográficos de indivíduos com câncer de boca no Brasil e o intervalo entre o diagnóstico e o início do tratamento.

## Métodos

### Delineamento

Trata-se de estudo transversal com dados secundários dos casos de câncer de boca do Brasil registrados entre 2011 e 2020.

### Participantes

A população de estudo foi composta por pessoas de ambos os sexos, de todas as idades, com diagnóstico confirmado de câncer de boca, classificados como C00 a C06, conforme a 10ª revisão da Classificação Internacional de Doenças – CID-10 (12).

Foram excluídos casos que não continham todas as variáveis necessárias para o estudo e casos com intervalo de tempo negativo entre diagnóstico e tratamento, indicando que a cirurgia precedeu o diagnóstico anatomopatológico, não sendo adequados à análise proposta nesta pesquisa.

### Variáveis

As variáveis dependentes do estudo foram intervalo entre diagnóstico e tratamento, correspondendo ao número de dias entre a data do diagnóstico (confirmação histopatológica) e a data do início do tratamento do câncer, e estadiamento do câncer no diagnóstico, determinado segundo o sistema tumor, nódulo regional ou metástase (TNM) de classificação dos tumores malignos (13). O intervalo foi categorizado em dias: 0-60, 61-90 e ≥91. De acordo com combinações das categorias T, N e M, o estadiamento foi categorizado em graus 0 (in situ), I, II, III e IV (13). 

As variáveis independentes foram sexo (masculino e feminino); idade (contínua e faixa etária, em anos [0-39, 40-79 e 80 ou mais]); raça/cor da pele (brancos, negros e outros); escolaridade (menor que 8 anos, 8 anos, 11 anos e 15 anos ou mais); estado conjugal (solteiro, casado/união consensual, viúvo e separado); custeio do tratamento (Sistema Único de Saúde [SUS] e plano de saúde/particular); e região (Norte, Nordeste, Sudeste, Sul e Centro-Oeste).

As variáveis categóricas foram descritas por meio de frequências absolutas e relativas. Para a variável idade (contínua), foram calculados a média, a mediana e o desvio-padrão.

### Fontes de dados e mensuração

Foram utilizados dados do Sistema de Informação de Registros Hospitalares de Câncer do Instituto Nacional de Câncer (Sis-RHC/INCA), que inclui características sociodemográficas, clínicas e do tratamento (https://irhc.inca.gov.br/RHCNet/selecionaTabulador.action?initial=1&local=todosho_sp&unidFed=) (14). 

Essa base de dados foi escolhida por proporcionar, simultaneamente, variáveis que permitiram a análise da interseccionalidade entre as vulnerabilidades sociais e do câncer de boca (15). 

### Controle de viés

A completude e a consistência dos registros, identificando dados faltantes e incoerências temporais, foram verificadas para a análise da qualidade dos dados. 

### Tamanho do estudo

Considerou-se a totalidade de registros disponíveis para o período, garantindo a representatividade da amostra. Essa abordagem evitou a amostragem aleatória, já que os dados eram secundários e abrangiam universo significativo da população-alvo.

### Métodos estatísticos

A associação entre cada variável independente e o intervalo entre diagnóstico e tratamento foi verificada pelo teste do qui-quadrado de Pearson. Posteriormente, aplicou-se a regressão logística multinomial para estimar a força de associação de cada variável isolada como preditor independente, tomando a categoria tempo de tratamento de 0 a 60 dias como referência.

Os perfis foram delineados por meio da análise de aglomerados, para agrupar casos ou objetos de acordo com o grau de semelhança observado entre eles. Foram utilizadas variáveis sociodemográficas, capazes de descrever agrupamentos de indivíduos internamente homogêneos e diferentes entre si, segundo estas características. No modelo, foram utilizadas as variáveis: sexo, idade, raça/cor da pele, escolaridade, custeio do tratamento, situação conjugal e regionalidade. 

O método utilizado foi o aglomerado em duas etapas, disponível no Statistical Package for Social Science for Windows, Incorporation, United States of America (SPSS 23.0), indicado para análise de dados cujas variáveis sejam contínuas e categóricas. 

Seu funcionamento é baseado em uma sequência de partições aglomerativas. No primeiro passo, foram formados os pré-aglomerados, que poderiam ser casos individuais ou pequenos grupos. No segundo passo, os grupos pré-aglomerados foram reagrupados, formando subperfis finais, segundo um número ideal de agrupamentos. Para a definição do melhor número de aglomerados, foi aplicado o critério de informação bayesiano (BIC), e a medida de distância utilizada foi o log-verossimilhança, ambas opções-padrão do programa.

Os perfis sociodemográficos foram descritos e comparados em relação às variáveis intervalo entre diagnóstico e tratamento e estadiamento do câncer no diagnóstico, por meio da análise de diferença de proporções usando-se o qui-quadrado de Pearson ou o teste exato de Fisher com correção de Bonferroni, ao nível de significância de 0,05.

A análise de regressão logística multinomial foi utilizada para verificar a força da associação entre os perfis delineados e a variável intervalo entre diagnóstico e tratamento, independentemente do estadiamento do diagnóstico, ao nível de significância de 0,05. A razão de chances (*odds ratio*, OR) foi calculada para quantificar a força das associações, juntamente com os intervalos de confiança de 95% (IC95%) para avaliar a precisão das estimativas. 

A categoria tempo de tratamento de 0 a 60 dias foi utilizada como referência para a análise de regressão, por corresponder ao prazo máximo para o início do tratamento, conforme preconizado pela Lei n^o^ 12.732/2012 (6).

### Acesso aos dados e métodos de limpeza

O banco de dados utilizado para análise, bem como códigos e demais dados pertinentes, estão disponíveis no repositório SciELO Data (https://doi.org/10.48331/scielodata.PLDF5O) (16). Para a tabulação dos dados, foi utilizado o programa Microsoft Excel.

### Pareamento dos dados

O estudo utilizou uma única base de dados, não havendo necessidade de pareamento com outras fontes. Foi feita a validação de integridade dos dados internamente por revisão manual de amostras aleatórias. As variáveis foram formatadas, padronizadas e categorizadas conforme critérios do estudo, agrupando-se as variáveis categóricas para uniformização e consistência na análise estatística.

## Resultados

No Sis-RHC/INCA, foram identificados no total 67.257 registros de câncer de boca. Foram inicialmente excluídos 13.358 casos (19,9%) sem informação sobre o intervalo entre diagnóstico e tratamento, restando 53.899 casos (80,1%). Em seguida, removeram-se 2.890 casos (4,3%) com intervalo negativo, totalizando 51.009 casos (75,8%) restantes. Por fim, 25.840 casos (38,4%) com ausência de, ao menos uma variável independente, foram excluídos, resultando em 25.169 casos (37,4%) incluídos na análise (Figura Suplementar 1).

A média de idade dos 25.169 casos foi de 60,1 anos, com desvio-padrão de 12,7 anos, e a mediana foi de 60 anos. Predominaram os indivíduos com as seguintes características: 40 a 79 anos (88,5%), sexo masculino (75,3%), raça/cor da pele negra (56,2%), escolaridade menor que 8 anos (66,1%), região Sudeste (37,6%), custeio do tratamento pelo SUS (85,8%) e estado conjugal casado/união consensual (50,2%). Menos da metade das pessoas com câncer de boca (43%) apresentou intervalo de até 60 dias entre diagnóstico e tratamento, e a maioria dos casos foi diagnosticada com o câncer em estágio grau IV (37.1%) (Tabela 1). A média e a mediana do intervalo entre diagnóstico e tratamento foram 95,9 e 70 dias, respectivamente.

**Tabela 1:** Características sociodemográficas e clínico-assistenciais dos pacientes com câncer de boca. Brasil, 2011-2020 (n=25.169)

Variáveis	n (%)
**Faixa etária** (anos)	
0-39	1.138 (4,6)
40-79	22.290 (88,5)
80 ou mais	1.741 (6,9)
Sexo	
Masculino	18.957 (75,3)
Feminino	6.212 (24,7)
**Raça/cor da pele**	
Brancos	10.849 (43,1)
Negros	14.143 (56,2)
Outros	177 (0,7)
**Escolaridade**	
Menor que 8 anos	16.636 (66,1)
8 anos	4.382 (17,4)
11 anos	3.385 (13,4)
15 anos ou mais	766 (3,0)
Região	
Norte	1.128 (4,5)
Nordeste	7.618 (30,3)
Sudeste	9.465 (37,6)
Sul	6.355 (25,2)
Centro-Oeste	603 (2,4)
**Custeio do tratamento**	
SUS	21.589 (85,8)
Plano de saúde/particular	3.580 (14,2)
**Estado conjugal**	
Solteiro	7.376 (29,3)
Casado/união consensual	12.628 (50,2)
Viúvo	2.777 (11,0)
Separado	2.388 (9,5)
**Intervalo entre diagnóstico e tratamento** (dias)	
0-60	10.826 (43,0)
61-90	5.001 (19,9)
≥91	9.342 (37,1)
**Estadiamento do câncer no diagnóstico**	
Grau 0 (in situ)	23 (0,1)
Grau I	1.372 (5,5)
Grau II	2.021 (8,0)
Grau III	3.127 (12,4)
Grau IV	9.348 (37,1)
Não informado	9.278 (36,9)

Todas as variáveis analisadas individualmente se mantiveram associadas com o intervalo de tempo entre diagnóstico e tratamento (Tabela 2). Ao se observarem as chances de início do tratamento com 91 dias ou mais, para cada uma das variáveis independentes, identificou-se que: homens apresentaram 7,8% menos chance de atraso em comparação às mulheres (OR 0,92; IC95% 0,86; 0,99); indivíduos com idade de 0 a 39 anos tiveram 18,3% menos chance em relação aos de 40 a 79 anos (OR 0,82; IC95% 0,71; 0,94); brancos, 10,9% menos chance em comparação aos negros (OR 0,89; IC95% 0,83; 0,96); pessoas com 15 anos ou mais de escolaridade, 36,6% menos chance em relação àquelas com menos de 8 anos (OR 0,63; IC95% 0,53; 0,76); residentes da região Sul, 52,3% menos chance quando comparados aos da região Sudeste (OR 0,48; IC95% 0,44; 0,51); e pacientes com custeio particular ou por plano de saúde apresentaram 27,6% menos chance frente àqueles com custeio pelo SUS (OR 0,72; IC95% 0,67; 0,79) (Tabela Suplementar 1).

**Tabela 2:** Associação entre as variáveis independentes e o intervalo de tempo entre diagnóstico e tratamento. Brasil, 2011-2020 (n=25.169)

Variáveis	0-60 dias n (%)	61-90 dias n (%)	≥91 dias n (%)	p-valor
**Faixa etária** (anos)				0,033
0-39	533 (46,8)	228 (20,0)	377 (33,1)	
40-79	9.532 (42,8)	4.420 (19,8)	8.338 (37,4)	
80 ou mais	761 (43,7)	353 (20,3)	627 (36,0)	
Sexo				0,001
Masculino	8.184 (43,2)	3.843 (20,3)	6.930 (36,6)	
Feminino	2.642 (42,5)	1.158 (18,6)	2.412 (38,8)	
**Raça/cor da pele**				<0,001
Brancos	5.157 (47,5)	1.983 (18,3)	3.709 (34,2)	
Negros	5.571 (39,4)	2.990 (21,1)	5.582 (39,5)	
Outros	98 (55,4)	28 (15,8)	51 (28,8)	
Escolaridade				<0,001
Menor que 8 anos	6.803 (40,9)	3.344 (20,1)	6.489 (39,0)	
8 anos	2.019 (46,1)	860 (19,6)	1.503 (34,3)	
11 anos	1.586 (46,9)	662 (19,6)	1.137 (33,6)	
15 anos ou mais	418 (54,6)	135 (17,6)	213 (27,8)	
Região				<0,001
Norte	434 (38,5)	198 (17,6)	496 (44,0)	
Nordeste	3.308 (43,4)	1.479 (19,4)	2.831 (37,2)	
Sudeste	3.379 (35,7)	2.135 (22,6)	3.951 (41,7)	
Sul	3.367 (53,0)	1.109 (17,5)	1.879 (29,6)	
Centro-Oeste	338 (56,1)	80 (13,3)	185 (30,7)	
**Custeio do tratamento**				<0,001
SUS	9.039 (41,9)	4.324 (20,0)	8.226 (38,1)	
Plano de saúde/particular	1.787 (49,9)	677 (18,9)	1.116 (31,2)	
**Estado conjugal**				<0,001
Solteiro	3.011 (40,8)	1.469 (19,9)	2.896 (39,3)	
Casado/união consensual	5.624 (44,5)	2.475 (19,6)	4.529 (35,9)	
Viúvo	1.173 (42,2)	550 (19,8)	1.054 (38,0)	
Separado	1.018 (42,6)	507 (21,2)	863 (36,1)	

No processo de aglomerado em duas etapas, as variáveis de maior importância na predição dos perfis foram: sexo, raça/cor da pele, escolaridade, estado conjugal, faixa etária e região. O custeio do tratamento apresentou o menor peso na formação dos perfis. Foram delineados quatro diferentes perfis (Tabela 3).

**Tabela 3:** Frequência das características sociodemográficas, segundo os perfis dos pacientes com câncer de boca. Brasil, 2011-2020 (n=25.169)

Características sociodemográficas	Perfil A 39,1% (9.843)	Perfil B 16,3% (4.108)	Perfil C 32,4% (8.150)	Perfil D 12,2% (3.068)
Sexo				
Masculino	81,6	76	78	47,4
Feminino	18,4	24	22	52,6
**Faixa etária** (anos)				
0-39	10	12	4	0,2
40-79	80	85	81	60
80 ou mais	10	3	15	39,8
**Raça/cor da pele**				
Brancos	96,9	1,65	0	40,7
Negros (pretos e pardos)	2,1	96,7	99,7	59,3
Outros	1	1,65	0,3	0
**Escolaridade** (anos)				
Menor que 8	59,7	0	100	85,1
8	18,2	53,6	0	13,9
11	15	40	0	1
15 ou mais	7,1	6,4	0	0
Região				
Norte	0,5	5,9	7	4,5
Nordeste	8,1	43,3	48,6	33
Sudeste	39,4	39,5	38	35,5
Sul	51,5	5,9	4	25
Centro-Oeste	0,5	5,3	2,4	2
**Estado conjugal**				
Solteiro	22,5	30	44,6	0
Casado/união consensual	61,4	50,2	55,4	0
Viúvo	0	8,8	0	84,3
Separado	16,1	11	0	15,7

Identificaram-se o perfil (A), de homens predominantemente brancos (96,9%) com escolaridade menor que 8 anos (59,7%), da região Sul (51,5%), casados ou em união consensual (61,4%), com idade entre 40 e 79 anos; perfil (B), de homens negros (96,7%), com escolaridade de 8 anos (53,6%), da região Nordeste (43,3%), casados ou em união consensual (50,2%) e com idade entre 40 e 79 anos; perfil (C), de homens negros (99,7%), com escolaridade menor que 8 anos (100%), da região Nordeste (48,6%), casados ou em união consensual (55,4%), com idade entre 40 e 79 anos; e por fim, perfil (D), de mulheres negras (59,3%), com escolaridade menor que 8 anos (85,1%), da região Sudeste (35,5%), viúvas (84,3%) e com idade entre 40 e 79 anos (Tabela 3).

A maioria dos indivíduos incluídos no perfil A (52,8%) apresentou intervalo entre diagnóstico e tratamento de 0 a 60 dias. Menos da metade dos indivíduos dos perfis B (43,7%), C (40,9%) e D (31,4%) iniciou tratamento neste intervalo. O perfil D se destacou por ter a maior proporção de pacientes (46,9%) com intervalo de tempo entre diagnóstico e tratamento ≥91 dias (p-valor<0,001) (Tabela 4).

**Tabela 4:** Comparação dos perfis sociodemográficos de pacientes com câncer de boca, segundo o intervalo de tempo entre diagnóstico e tratamento e o estadiamento. Brasil, 2011-2020 (n=25.169)

Características	Perfil A^a^	Perfil B^b^	Perfil C^c^	Perfil D^d^	p-valor
**Intervalo entre diagnóstico e tratamento** (dias)					<0,001
0-60	52,8	43,7	40,9	31,4	
61-90	16,8	23,7	19,7	21,7	
≥91	30,3	32,6	39,4	46,9	
Estadiamento					0,005
Graus 0 (in situ) e I	11,4	7,3	7,0	15,4	
Grau II	12,3	16,9	13,1	13,1	
Graus III e IV	76,3	75,8	79,9	71,5	

^a^Perfil A: homens, de 40 a 79 anos, brancos, com escolaridade menor que 8 anos, do Sul e casados ou em união consensual; ^b^Perfil B: homens, de 40 a 79 anos, negros, com escolaridade de 8 anos, do Nordeste e casados ou em união consensual; ^c^Perfil C: homens, de 40 a 79 anos, negros, com escolaridade menor que 8 anos, do Nordeste e casados ou em união consensual; ^d^Perfil D: mulheres, de 40 a 79 anos, negras, com escolaridade menor que 8 anos, do Sudeste e viúvas.

A proporção de indivíduos diagnosticados nos estágios III e IV do câncer foi significativamente alta em todos os perfis, sendo que os perfis A (76,3%), B (75,8%), C (79,9%) e D (71,5%) foram semelhantes entre si. Nos estágios iniciais, graus 0 e I, os perfis A (11,4%) e D (15,4%) apresentaram proporções maiores que os perfis B (7,3%) e C (7,0%). No estágio II, todos os perfis foram semelhantes, com proporções de 12,3% (A), 16,9% (B), 13,1% (C) e 13,1% (D) (p-valor 0,005) (Tabela 4).

Comparados ao perfil D, os indivíduos dos perfis A, B e C apresentaram diferentes chances de iniciar o tratamento no intervalo entre 61 e 90 dias e ≥91 dias. As associações entre os perfis B e C e o intervalo de 61 a 90 dias não foram estatisticamente significativas. No entanto, o perfil A manteve-se associado a menor chance de ter intervalo de 61 a 90 dias. Todos os perfis mantiveram-se associados ao intervalo ≥91 dias, sendo que o perfil A apresentou chance 61,5% menor de iniciar o tratamento com esse intervalo do que o perfil D (OR 0,38; IC95% 0,26; 0,55), o perfil B teve chance 50% menor (OR 0,50; IC95% 0,32; 0,77), e o perfil C teve chance 35,4% menor (OR 0,64; IC95% 0,45; 0,92) (Tabela 5).

**Tabela 5:** Razão de chances (*odds ratio*, OR) e intervalos de confiança de 95% (IC95%) do intervalo de tempo entre diagnóstico e tratamento, de acordo com os perfis de pacientes com câncer de boca. Brasil, 2011-2020 (n=25.169)

Perfis	61-90 dias OR (IC95%)	p-valor	≥91 dias OR (IC95%)	p-valor
Homens brancos de menor escolaridade da região Sul, com companheiro(a)	0,46 (0,29; 0,71)	0,001	0,38 (0,26; 0,55)	≤0,001
Homens negros de maior escolaridade da região Nordeste, com companheiro(a)	0,78 (0,47; 1,30)	0,348	0,50 (0,32; 0,77)	0,002
Homens negros de menor escolaridade da região Nordeste, com companheiro(a)	0,69 (0,45; 1,08)	0,107	0,64 (0,45; 0,92)	0,016
Mulheres negras de menor escolaridade da região Sudeste, viúvas	1,00	__	1,00	__

## Discussão

Este estudo teve como objetivo analisar desigualdades no tempo entre diagnóstico e início do tratamento do câncer de boca no Brasil, com ênfase em marcadores sociais da diferença sob a perspectiva da interseccionalidade. A análise interseccional revelou desigualdades no acesso: pacientes do perfil A (homens brancos, sulistas, com melhores condições socioeconômicas) iniciaram o tratamento mais rapidamente, enquanto os do perfil D (mulheres negras, da região Sudeste, com baixa escolaridade) tiveram maior chance de atraso.

No estudo em questão, foram identificadas diferenças estatísticas entre algumas características dos pacientes que permaneceram na análise final e dos que foram excluídos. O uso de um banco de dados com alta proporção de variáveis não preenchidas limita a generalização dos resultados, que devem ser atribuídos apenas à população analisada, não representando necessariamente todos os pacientes em tratamento de câncer de boca no Brasil, no período considerado (17). 

Verificou-se predominância de diagnósticos em estágios avançados da doença (III e IV), o que reduz as chances de sucesso no tratamento e piora o prognóstico (18). Esse padrão de diagnóstico tardio indica falhas no sistema de saúde em termos de triagem e detecção precoce, pois sugere que há insuficiência de programas de rastreamento e conscientização sobre o câncer de boca institucionalizados nacionalmente (19). Isso pode ser devido a vários desafios, como a carência de campanhas educativas, barreiras no acesso a serviços de saúde primários e a falta de treinamento adequado dos profissionais de saúde para reconhecer os sinais precoces da doença (19).

A demora no início do tratamento do câncer de boca é outro problema significativo, pois reduz as chances de cura, piora o prognóstico e eleva os custos para o sistema de saúde (7). Apesar da Lei n^o^ 8.080/1990, menos da metade dos pacientes iniciou o tratamento dentro dos 60 dias recomendados, o que mostra predominância ainda de atrasos no tratamento. Assim, essa demora reflete a desarticulação entre os níveis de atenção e as dificuldades logísticas de encaminhamento e reforça a necessidade de estratégias para fortalecer a linha de cuidado oncológica, com integração entre os serviços, vigilância ativa, regulação eficiente e expansão da capacidade nos centros de referência (20).

Observou-se maior prevalência de câncer de boca em homens, sugerindo influência do sexo na ocorrência da doença, possivelmente relacionada à maior exposição masculina a fatores de risco, como tabagismo, etilismo, condições ocupacionais adversas e menor adesão a práticas preventivas, influenciada por normas de masculinidade (4,21). Em relação à idade, a proximidade entre média e mediana (60 anos) aponta para distribuição simétrica, reforçando que o câncer de boca acomete principalmente pessoas em torno dos 60 anos, compatível com o perfil epidemiológico descrito na literatura, que associa o câncer de boca ao acúmulo de exposições ao longo da vida (4). Esse padrão etário reforça a importância de políticas públicas voltadas à prevenção, rastreamento e diagnóstico precoce na população idosa, especialmente em contextos de envelhecimento populacional, como o brasileiro (22). 

Cada variável de forma independente se mostrou preditora do tempo entre diagnóstico e início do tratamento do câncer de boca, com maior atraso observado em mulheres, pessoas de 40 a 79 anos, negros, indivíduos com baixa escolaridade, residentes da região Sudeste e pacientes com custeio pelo SUS. De forma semelhante, em coorte retrospectiva com mais de 79 mil casos de câncer colorretal (2006-2015), observou-se maior atraso entre pacientes com mais de 50 anos, negros e com baixa escolaridade (23). Do mesmo modo, estudo de coorte retrospectiva de 137.593 mulheres com câncer de mama cadastradas no Sis-RHC (2000-2011) apontou maior vulnerabilidade ao atraso entre idosas, não brancas, sem companheiro, com baixa escolaridade e atendidas pelo SUS (24). 

Contudo, embora as variáveis, de forma individual, hajam influenciado no tempo entre o diagnóstico e o início do tratamento do câncer de boca, é essencial analisar o impacto do perfil sociodemográfico no tempo até o início do tratamento, sob a perspectiva da interseccionalidade dessas variáveis independentes. Estudos de revisão apontam que políticas focadas em apenas uma dimensão da identidade (como sexo ou raça/cor da pele isoladamente) falham em captar a complexidade das vivências individuais, enquanto a incorporação da interseccionalidade permite o desenvolvimento de estratégias mais eficazes contra desigualdades sistêmicas (25). 

A análise interseccional revelou que mulheres negras, da região Sudeste, entre 40 e 79 anos, viúvas e com baixa escolaridade enfrentam maior demora no início do tratamento, indicando vulnerabilidades associadas à sobreposição de marcadores sociais. Esse padrão é compatível com o observado em estudo transversal semelhante realizado em Belo Horizonte com 715 mulheres com câncer de mama cadastradas no Sis-RHC (2010-2013), no qual o perfil de mulheres negras, com escolaridade inferior a 8 anos e tratamento via SUS também apresentou maior chance de iniciar o tratamento do câncer de mama após 91 dias, em comparação com mulheres brancas, mais escolarizadas e atendidas na rede privada (17). Esse resultado exibe que, quanto maior o perfil de vulnerabilidade social das mulheres, maior a chance de atraso (p-valor<0,001) (17). A variável estado conjugal apresentou importância igual a zero na predição dos perfis nesse estudo anterior (17), diferentemente do presente estudo, em que a variável que apresentou menor peso na formação dos perfis foi o custeio do tratamento. 

Essas vulnerabilidades sociais impactam de maneiras distintas o início do tratamento, de modo que, quando interseccionadas, culminam no atraso observado no perfil D. Homens brancos casados do Sul, por exemplo, que compõem o perfil A, tendem a ter acesso mais rápido ao tratamento, devido a melhores condições educacionais, menor discriminação e maior desenvolvimento do sistema de saúde regional (7). Em contraste, mulheres negras viúvas do Sudeste, que compõem o perfil D, enfrentam múltiplas barreiras decorrentes da interseccionalidade dessas variáveis, dificultando o acesso oportuno ao tratamento (17,26). A ausência de parceiro está associada a maiores atrasos no diagnóstico, possivelmente por menor acesso a cuidados preventivos e maior sobrecarga familiar e financeira (27). Já mulheres casadas tendem a buscar atendimento mais cedo, beneficiadas pelo apoio do parceiro na identificação e discussão dos sintomas (28). Níveis mais altos de escolaridade também favorecem a comunicação com profissionais de saúde e a descrição dos sintomas, facilitando a trajetória no sistema de saúde para o diagnóstico e o início do tratamento (29).

Este estudo possui algumas limitações a serem consideradas. A natureza agregada dos dados limita a individualização das análises e impede inferências causais (18). Ressalta-se também que este é um dos primeiros estudos a abordar o intervalo entre diagnóstico e tratamento do câncer de boca sob a perspectiva interseccional, destacando-se a necessidade de futuras investigações com outras bases e recortes regionais, para se aprofundar a compreensão das vulnerabilidades sociais envolvidas (7). Fatores clínicos potencialmente confundidores, como infecção pelo papilomavírus humano tipo 16 (HPV-16), exposição solar e obesidade, não foram considerados, por ausência dessas informações na base de dados. Uma limitação importante refere-se à qualidade do preenchimento dos registros no Sis-RHC/INCA, com subnotificações e discrepâncias regionais que podem comprometer a precisão das estimativas, influenciadas por fatores como infraestrutura, capacitação e padronização dos registros (30), o que leva à necessidade de análise cautelosa dos achados do estudo. Ainda assim, a qualidade dos dados tem melhorado ao longo dos anos, reforçando a importância de seu aprimoramento contínuo para a vigilância epidemiológica do câncer e o planejamento da atenção oncológica (17,30). 

A análise dos perfis sociodemográficos mostra que a interseccionalidade aprofunda a compreensão das desigualdades no acesso à saúde, destacando a necessidade de políticas que considerem essas interações para ampliar o acesso oportuno ao tratamento oncológico de grupos vulnerabilizados e garantir a aplicação dos princípios de integralidade, universalidade e equidade do SUS à realidade de cada indivíduo (7).

Conclui-se que mulheres negras, da região Sudeste, com baixa escolaridade, entre 40 e 79 anos e viúvas iniciam o tratamento, em sua maioria, após 91 dias do diagnóstico. Apesar de a maior parte dos casos serem diagnosticados nos estágios III e IV, há desigualdades significativas na forma como diferentes grupos sociodemográficos acessam e iniciam o tratamento do câncer de boca. 

## Data Availability

Todo o conjunto de dados que dá suporte aos resultados deste estudo foi disponibilizado no SciELO Data e pode ser acessado em https://doi.org/10.48331/scielodata.PLDF5O.

## Material suplementar

Figura suplementar 1, Tabela Suplementar 1.
